# Diet quality determinants among manufacturing workers in Brazil: Socio-demographic factors, food choices, and ultra-processed foods

**DOI:** 10.1371/journal.pone.0349760

**Published:** 2026-06-17

**Authors:** Anissa Melo Souza, Karina Gomes Torres, Raiane Medeiros Costa, Gabriela Santana Pereira, Célia Márcia Medeiros Morais, Antonio Gouveia Oliveira, Ingrid Wilza Leal Bezerra

**Affiliations:** 1 Postgraduate Program in Health Sciences, Federal University of Rio Grande do Norte, Natal, Rio Grande do Norte, Brazil; 2 Nutrition Department, Health Science Center, Federal University of Rio Grande do Norte, Natal, Rio Grande do Norte, Brazil; 3 Pharmacy Department, Health Science Center, Federal University of Rio Grande do Norte, Natal, Rio Grande do Norte, Brazil; University of Georgia, UNITED STATES OF AMERICA

## Abstract

This study evaluated the association between diet quality and socio-demographic factors, anthropometric measures, food choices, and consumption of processed foods in a low-income Brazilian manufacturing worker population, addressing gaps in evidence from low- and middle-income countries (LMICs). A cross-sectional study was conducted with 921 workers from 33 industries in Rio Grande do Norte, Brazil, selected via stratified proportional and multi-stage sampling. Diet quality was assessed using the Diet Quality Index-International (DQI-I), which evaluates variety, adequacy, moderation, and balance. Dietary intake was measured via 24-hour recalls, and foods were classified by processing level (NOVA system). Socio-demographic, anthropometric, and food choice data (using the Food Choice Questionnaire) were collected. Associations were analyzed using multilevel linear regression. Higher diet quality was associated with being male (p = 0.001). Among women, diet quality improved with age (p = 0.018) and participation in a food assistance program (p < 0.001), whereas those variables showed no association among men. Prioritizing natural food content predicted higher DQI-I scores in both sexes (p < 0.05), while weight control was significant only for women (p = 0.034). Unprocessed food intake showed a positive association (p < 0.001) with diet quality, whereas ultra-processed food consumption was inversely associated (p < 0.001), most notably with diet variety and adequacy in both sexes, and with moderation in women. Food choices also influence diet quality, with people who value health benefits, natural content, weight control, and familiar products being more likely to consume diets with better variety and adequacy. No significant associations were found between diet quality and anthropometric measures. Socio-demographic factors, food choices, and levels of food processing significantly influence diet quality in this low-income Brazilian population. Public health strategies targeting reductions in ultra-processed food consumption and supporting food assistance programs may improve dietary patterns, particularly among women. These findings highlight the need for context-specific interventions to address nutrition transitions in LMICs.

## Introduction

Diet quality is a multidimensional construct that reflects the healthfulness and nutritional adequacy of an individual’s dietary intake, based on the diversity and proportionality of foods consumed in relation to dietary guidelines and health outcomes [1]. Unlike approaches focused on isolated nutrients or food groups, diet quality assessments evaluate overall dietary patterns, acknowledging that foods are consumed in combination and exert synergistic effects on health [2]. A high-quality diet is typically characterized by abundant consumption of fruits, vegetables, whole grains, lean proteins, and healthy fats, while minimizing processed foods, added sugars, sodium, and unhealthy fats [3]. Given the growing global burden of diet-related chronic diseases, understanding and measuring diet quality has become a critical focus of nutritional epidemiology and public health research [4].

To monitor and compare diet quality, several indices have been developed based on adherence to dietary guidelines or evidence-based recommendations. Common indices include the Healthy Eating Index (HEI), the Alternative Healthy Eating Index (AHEI), the Mediterranean Diet Score (MDS), and the Diet Quality Index-International (DQI-I) [5]. The DQI-I is particularly useful for cross-national comparisons, as it was designed to be applicable across diverse cultural and dietary contexts and captures both nutrient deficiencies and excesses, allowing for comprehensive analysis of dietary imbalances [6]. The assessment of diet quality involves several key dimensions, including variety, adequacy, moderation, and balance [5]. Variety refers to the diversity of foods consumed across and within food groups, ensuring a broad spectrum of essential nutrients [7]. Adequacy assesses whether an individual’s diet meets recommended intake levels for essential nutrients, such as vitamins, minerals, fiber, and protein [8]. Moderation evaluates the consumption of foods and nutrients that should be limited, such as saturated fats, trans fats, added sugars, and sodium [9]. Finally, balance considers the proportionality of macronutrients (carbohydrates, fats, and proteins) and the overall distribution of food groups to ensure a nutritionally optimal diet [10]. These dimensions collectively contribute to a comprehensive understanding of dietary patterns and their potential health impacts.

Numerous studies have linked higher diet quality to better nutrient adequacy and a reduced risk of chronic diseases. Diets rich in minimally processed foods provide essential vitamins, minerals, fiber, and bioactive compounds that support metabolic function and reduce inflammation [11]. The Mediterranean and DASH (Dietary Approaches to Stop Hypertension) dietary patterns, both considered high-quality, are associated with improved intake of beneficial nutrients and lower consumption of harmful dietary components [12,13].

Conversely, poor diet quality is a leading global risk factor for non-communicable diseases. The Global Burden of Disease Study identified suboptimal diets as a more significant contributor to mortality than tobacco use or physical inactivity [14]. Diets low in whole grains, fruits, nuts, and seeds, and high in sodium, processed meats, and trans fats contribute substantially to disease burden.

Diet quality is shaped by a complex interplay of socioeconomic, demographic, cultural, and environmental factors. Socioeconomic status is one of the strongest determinants; individuals with higher income and education tend to consume more nutrient-dense foods [15]. In contrast, those with limited resources often rely on energy-dense, nutrient-poor diets due to financial constraints and poor access to healthy options [16]. Age and gender also influence diet quality, older adults and women typically report healthier diets, potentially due to greater health awareness and involvement in food preparation [17,18]. Cultural dietary patterns further influence diet quality, with traditional diets generally offering more nutritional value than westernized, ultra-processed food patterns [19].

The food environment, which encompasses the physical, economic, political, and sociocultural contexts in which consumers interact with food, in areas with low geographic availability of food stores, and public policies also affect dietary behaviors. While the former is associated with lower diet quality [20], policy interventions such as front-of-pack labelling, taxes on sugar-sweetened beverages, and nutrition assistance programs have shown promise in improving population-level dietary patterns [21].

Although several systematic reviews have investigated the determinants of diet quality [15], most focus on high-income countries. There is a critical need for more evidence from low- and middle-income countries, particularly in contexts marked by the coexistence of undernutrition and obesity, a phenomenon known as the double burden of malnutrition. In Brazil, this paradox is especially evident among low-income groups and is exacerbated by the country’s ongoing nutrition transition, characterized by a shift from traditional diets to processed, energy-dense foods [22].

Investigating diet quality in low-income Brazilian populations offers valuable insights for public health nutrition. Identifying associations between diet quality, socio-demographic factors, anthropometric indicators, and the consumption of processed and ultra-processed foods can guide targeted interventions and inform strategies to promote healthier food environments. Moreover, such research contributes to the global understanding of nutritional transitions in urbanizing, low-income settings. Furthermore, manufacturing workers in middle-income countries often occupy a unique nutritional transition space, being particularly vulnerable to malnutrition. This vulnerability is driven by several factors: long working hours that limit time for meal preparation, the pervasive availability of cheap, ultra-processed foods in and around industrial zones, and socio-economic constraints that can make nutrient-dense options less accessible. Understanding the specific dietary determinants for this group has significant economic and social ramifications, as population constitutes the backbone of the industrial sector, and their health and productivity are directly linked to the nation’s economic output. Poor diet quality among these workers leads to a higher prevalence of non-communicable diseases that not only diminishes their quality of life but also results in increased absenteeism, reduced productivity, and higher healthcare costs for both individuals and the public health system.

Few studies have simultaneously examined diet quality in relation to food choice motivations and the consumption of ultra-processed foods within the same analytical framework. This study aims to address this gap by focusing on an economically relevant and understudied population, while integrating multiple dimensions of dietary assessment, including diet quality, food choice motivations, and food processing level. Therefore, the aim of this study was to evaluate the association of diet quality with socio-demographic variables, anthropometric measures, individual food choices, and the consumption of processed and ultra-processed foods in a representative sample of a low-income manufacturing workers from Brazil. The findings can directly inform targeted workplace wellness initiatives, strengthen public policies aimed at improving food environments, and guide interventions that not only enhance worker well-being but also contribute to a more robust and sustainable economy.

## Materials and methods

### Study design and population

This observational, cross-sectional study employed prospective selection and direct interviews to evaluate manufacturing workers across the state of Rio Grande do Norte in northeastern Brazil. A representative sample was obtained through a combined stratified proportional and multi-stage sampling strategy. Factories were stratified by three economic sectors with greater representativeness in the state (food/beverages, non-metallic minerals, and textiles), by size (small: < 50 workers; medium: 50–500; large: > 500), and by participation in the Workers’ Food Program (WFP), a Brazilian public policy of food and nutritional assistance through which participating companies offer in-house meals in exchange for income tax benefits [23].

In the first stage, factories were selected from a grid provided by the state’s industrial federation using stratified random sampling in proportion to the number of factories in each stratum, with written consent obtained from all sampled factories. Workers within each selected factory were then chosen through simple random sampling from HR-provided employee lists. Eligible participants included adults over 18 years of age who provided written consent; temporary workers and pregnant women were excluded.

The study adhered to the Helsinki Declaration and received ethical approval from Hospital Universitário Onofre Lopes (Authorization 2.198.545/2017). Data collection involved demographic (age, sex, marital status), socioeconomic (education, income), and anthropometric measures (weight, height, waist circumference). Weight was measured using a Tanita (Tanita Corp, Tokyo, Japan) InnerScan digital scale (±0.05 kg precision), height with a portable Sanny stadiometer (Sanny, São Bernardo do Campo-SP, Brazil), and waist circumference with a Cescorf flexible steel tape (Cescorf, Porto Alegre-RS, Brazil). Body Mass Index (BMI) was calculated as (weight in kg)/(height in metres)², and cardiovascular risk was assessed using sex-specific waist circumference thresholds.

The physical activity level was assessed using the short version of the International Physical Activity Questionnaire (IPAQ) and expressed as metabolic equivalents of task (METs) per week. MET-minutes/week were calculated by multiplying the frequency (days/week), duration (minutes/day), and the standard MET values assigned to each activity intensity, as follows: 3.3 METs for walking, 4.0 METs for moderate-intensity activities, and 8.0 METs for vigorous-intensity activities. The total physical activity score was obtained by summing the MET-minutes/week for walking, moderate, and vigorous activities.

Dietary intake was assessed using the 24-hour recall method (24HR), based on the USDA 5-step method to minimize bias. Participants reported all foods and beverages consumed in the previous 24 hours, including preparation methods and quantities. To reflect habitual intake, data were collected from Tuesday to Saturday. Food amounts were converted to grams or millilitres using direct weighing, photographic records, food labels, and standardised manuals. Nutrient analysis provided daily energy (kcal), macronutrient (g, % energy), and micronutrient (mg, % RDA) intakes using reference tables.

Diet quality was assessed using the DQI-I, which evaluates four domains of a healthy diet: Variety (diversity of main food groups and protein sources), Adequacy (diet components providing essential nutrients such as fruits, vegetables, grains, fiber, vitamin C, iron, and calcium), Moderation (limits on diet components associated with poor health outcomes, including total and saturated fat, cholesterol, sodium, and empty-calorie foods), and Balance (proportion between macronutrients and fatty acid composition). Empty-calorie foods were defined as those with low nutrient density (nutrient-to-energy ratio <1 per 100 g) across 14 categories.

Dietary determinants were evaluated using the validated Brazilian Portuguese version of the Food Choice Questionnaire (FCQ) [24], which quantifies the importance given to nine dimensions: health, mood, convenience, sensory appeal, natural content, price, weight control, familiarity, and ethical concerns; through 36 items, with higher scores representing greater importance. Foods were classified both by processing level, using the NOVA [25] classification system (unprocessed, culinary ingredients, processed, ultra-processed), and by similarity in nutritional value, composition, and origin, which aligned with the DQI-I matrix.

### Statistical analysis

Sample size calculation was based on the 30-by-30 two stage cluster sampled design method widely used in population surveys. The sampling plan defined a selection of 30 companies for the first stage and 30 workers randomly sampled within in of those companies, resulting in a target sample size of 900 workers.

Statistical analyses present descriptive data as means ± SD (standard deviation) or counts (%). As the DQI-I was normally distributed (p = 0.10 by the Shapiro–Wilk test), multilevel linear regression was used to analyse variables associated with DQI-I scores. The nine sampling strata and an indicator variable representing participation in the WFP were included as fixed-effect covariates, and company was included as a random effect to account for clustering of workers within companies. To account for the differential physical requirements among jobs and for overall energy consumption, which might affect appetite and food preferences, all analyses were adjusted by level of physical activity expressed as metabolic equivalents of task (METs) per minute per week, and for total daily energy intake. Results are presented as least squares mean (regression coefficients) with 95% confidence intervals (CI). Robust variance estimates were used to account for eventual model misspecification. Statistical significance was set at *p* < 0.05. Analyses were conducted using Stata 15 (StataCorp., College Station, TX, USA). The study data is available in S1 File of the Supporting Information.

## Results

Between September 2017 and July 2018, 921 workers from 33 manufacturing industries were evaluated: 515 (55.9%) were men, with a mean age of 38.2 ± 10.7 years, a mean of 10.5 ± 3.58 years of schooling, and a mean income of 1.45 ± 1.61 minimum wages; 577 (62.7%) were married, and 638 (69.3%) had children. The distribution of sampled industries by economic sector included 14 in food/beverages, 6 in non-metallic minerals, and 13 in textiles. By company size, 14 were small, 13 medium, and 6 large.

Workers’ sex was associated with diet quality, as measured by the DQI-I scale, with males exhibiting higher scores than females (difference: 2.999, 95% CI: 1.201 to 4.796, *p* = 0.001). Among other demographic variables (Table 1), age was significantly associated with diet quality in females (*p* = 0.018), with older age correlating with higher DQI-I scores, suggesting that diet improves with age in women. In contrast, no such trend was observed in males (*p* = 0.26). Participation in food assistance programs was linked to improved diet quality in females (*p* < 0.001), but such an association was not observed in males (*p* = 0.73). Other demographic factors, including marital status, number of children, education, and income, showed no significant associations with diet quality in either sex.

**Table 1:** Association between diet quality (DQI-I scores) and demographic variables.

Variable	Women (n = 406)			Men (n = 515)		
	m	95%CI	p	m	95%CI	p
Age, years	0.114	0.195;0.207	**0.018**	0.031	–0.023;0.085	0.26
Married	0.303	–1.439;2.,46	0.73	0.434	–0.523;1.390	0.37
Children	0.752	–1.617;3.122	0.53	0.879	–0.477;2.236	0.20
Number of children	–0.235	–0.826;0.357	0.44	0.281	–0.107;0.669	0.16
Education, years	–0.304	–1.330;0.721	0.56	–0.480	–1.134;0.173	0.15
Income, minimum wages	0.490	–,.956;1.937	0.51	–0.182	–0.4341;0.070	0.16
Participant in WFP	3.078	1.414;4.742	**<0.001**	0.336	–1.532;2.203	0.73

m: least squares mean (regression coefficient); CI: confidence interval; bold p-values are statistically significant.

When examining anthropometric and cardiovascular risk factors (Table 2), the study found no significant associations between DQI-I scores and BMI, waist circumference, and cardiovascular risk in either sex. However, overweight status was negatively associated with DQI-I scores but only in males (p = 0.044).

**Table 2:** Association between diet quality (DQI-I scores) and anthropometric measures, nutritional status, and cardiovascular risk.

Variable	Women (n = 406)			Men (n = 515)		
	m	95%CI	p	m	95%CI	p
Body Mass Index (kg/m²)	–0.036	–0.187;0.115	0.64	–0.049	–0.234;0.136	0.60
Waist circumference (cm)	0.006	–0.057;0.069	0.84	–0.033	–0.097;0.031	0.31
Increased cardiovascular risk	0.393	–0.804;1.591	0.52	0.288	–1.120;1.696	0.69
Overweight (BMI > 25 kg/m^2^)	–0.804	–2.079;0.470	0.64	–1.201	–2.371;–0.031	**0.044**

m: least squares mean (regression coefficient); CI: confidence interval.

The analysis of dietary choice dimensions (Table 3) revealed that prioritizing natural food content was associated with higher DQI-I scores in both sexes (p = 0.033 in females and p = 0.021 in males). Additionally, females who considered weight control in their food choices had significantly better diet quality (p = 0.034), whereas no such association was observed in males (p = 0.32). Other dimensions, such as health, mood, convenience, and sensory appeal, showed no significant associations with diet quality.

**Table 3:** Association between diet quality (DQI-I scores) and dimensions of food choices.

FCQ dimension	Women (n = 406)			Men (n = 515)		
	m	95%CI	p	m	95%CI	p
Health	0.670	–0.634;1.973	0.31	0.312	–0.632;1.255	0.52
Mood	–0.162	–0.993;0.669	0.70	0.029	–0.675;0.734	0.94
Convenience	0.609	–0.485;1.704	0.28	–0.085	–0.904;0.735	0.84
Sensory appeal	0.497	–1.339;2.332	0.60	–0.066	–0.936;0.803	0.88
Natural content	1.132	0.092;2.173	**0.033**	0.667	0.102;1.232	**0.021**
Price	0.333	–0.894;1.560	0.60	0.111	–0.956;1.178	0.84
Weight control	0.954	0.071;1.836	**0.034**	0.420	–0.415;1.254	0.32
Familiarity	0.164	–1.039;1.367	0.79	0.152	–0.775;1.078	0.75
Ethical concern	0.048	–0.826;0.921	0.92	–0.153	–0.934;0.627	0.70

m: least squares mean (regression coefficient); CI: confidence interval. Bold p-values are statistically significant.

The strongest determinants of diet quality emerged from the analysis of the degree of food processing (Table 4). Higher consumption of unprocessed foods was strongly associated with better DQI-I scores in both sexes (*p* < 0.001 in both), while lower intake of ultra-processed foods was similarly associated with improved diet quality (*p* < 0.001 in both). Consumption of processed foods also showed a negative association with diet quality (*p* < 0.001 in both).

**Table 4:** Association between diet quality (DQI-I scores) and consumption of NOVA food categories as percentage of total energy intake.

NOVA food categories	Women (n = 406)			Men (n = 515)		
	m	95%CI	p	m	95%CI	p
Unprocessed	0.231	0.189;0.273	**<0.001**	0.170	0.123;0.217	**<0.001**
Culinary ingredients	–0.323	–0.535;–0.111	**0.003**	–0.295	–0.485;–0.104	**0.002**
Processed	–0.171	–0.233;–0.110	**<0.001**	–0.094	–0.147;–0.040	**<0.001**
Ultra-processed	–0.152	–0.211;–0.084	**<0.001**	–0.104	–0.163;–0.046	**<0.001**

m: least squares mean (regression coefficient); CI: confidence interval. Bold p-values are statistically significant.

Table 5 presents a granular analysis of the DQI-I components in relation to ultra-processed food consumption. Negative associations were observed in the “Variety” and “Adequacy” components for both sexes, and in the Moderation component in females.

**Table 5:** Association of ultra-processed food consumption as percent of total daily energy intake with diet quality (DQI-I scores).

DQI-I component	Women (n = 406)			Men (n = 515)		
	m	95%CI	p	m	95%CI	p
Variety	–0.067	–0.081;–0.054	**<0.001**	–0.050	–0.069;–0.033	**<0.001**
Adequacy	–0.037	–0.057;–0.016	**<0.001**	–0.046	–0.075;0.017	**0.002**
Moderation	–0.048	–0.084;–0.011	**0.011**	–0.021	–0.051;–0.010	0.18
General balance	–0.002	–0.010;0.007	0.72	0.012	–0.001;0.026	0.07

m: least squares mean (regression coefficient); CI: confidence interval. Bold p-values are statistically significant.

Table 6 provides a more detailed understanding of the role of ultra-processed foods in shaping diet quality, linking food choice motivations to specific dimensions of the DQI-I, with adjustment for worker’s sex. The “Health” dimension was positively associated with the “Variety” (p = 0.014) and “Adequacy” (p = 0.006) components of the DQI-I; “Natural content” was positively associated with the “Variety” (p = 0.032), “Adequacy” (p = 0.003), and “Moderation” (p = 0.042); “Weight control” was associated with the “Variety” (p < 0.001) and “Adequacy” (p = 0.007); “Familiarity” was positively associated with “Variety” (p = 0.28).

**Table 6:** Association of FCQ dimensions with components of the DQI-I.

FCQ dimensions	DQI-I components							
	Variety		Adequacy		Moderation		General balance	
	m	p	m	p	m	p	m	p
Health	0.225	**0.014**	0.416	**0.006**	–0.134	0.61	–0.007	0.95
Mood	0.053	0.48	0.060	0.66	–0.202	0.20	0.077	0.19
Convenience	–0.031	0.75	–0.060	0.70	0.259	0.25	–0.006	0.95
Sensory appeal	1.116	0.26	0.220	0.35	–0.173	0.58	–0.154	0.26
Natural content	0.157	**0.032**	0.307	**0.003**	0.348	**0.042**	0.096	0.19
Price	–0.135	0.25	–0.121	0.45	0.498	0.08	0.036	0.75
Weight control	0.263	**<0.001**	0.414	**0.007**	0.036	0.85	0.029	0.68
Familiarity	0.182	**0.028**	0.151	0.39	–0.223	0.25	–0.051	0.42
Ethical concern	0.135	0.11	0.141	0.41	–0.133	0.49	–0.135	0.07

m: least squares mean (regression coefficient). Bold p-values are statistically significant.

## Discussion

The present study aimed to evaluate the association between diet quality and socio-demographic variables, anthropometric measures, individual food choices, and the consumption of processed and ultra-processed foods in a Brazilian manufacturing workers population. The results offer a novel perspective on the determinants of diet quality in low- and middle-income countries (LMICs) and highlight the complex interplay of socio-economic, behavioral, and environmental factors influencing dietary patterns. Several key determinants of diet quality were identified, including sex, age, participation in food assistance programs, food choice dimensions (particularly natural content and weight control), and the level of food processing. Notably, higher consumption of unprocessed foods and lower intake of ultra-processed foods were strongly associated with better diet quality, as measured by the DQI-I.

The observed statistically significant difference in DQI-I scores between males and females in this low-income Brazilian population, with males exhibiting better diet quality, may be attributed to a combination of sociocultural, economic, and behavioral factors. In many low- and middle-income settings, gender roles influence dietary patterns, with males often prioritized in household food allocation due to traditional perceptions of men as primary breadwinners requiring greater caloric and nutrient intake [26], while women may compromise their own dietary quality to ensure adequate nutrition for male family members. Additionally, socioeconomic pressures may lead women to adopt food coping strategies, such as meal skipping or reliance on cheaper, less nutritious foods, to manage household budgets [27]. In some Brazilian communities, traditional masculinity is also associated with higher meat consumption, which, while not inherently healthier, may contribute to greater protein intake and dietary variety in men compared to women, who may consume more carbohydrates and lower-cost staples [28]. These findings align with broader literature indicating that gender intersects with socioeconomic status to shape dietary behaviors, often disadvantaging women in resource-limited settings [29].

Age was another significant factor, particularly among women; older age was associated with higher diet quality. This finding is consistent with studies suggesting that older adults often display more stable and health-conscious dietary patterns [17], potentially due to the consolidation of well-established cultural and regional habits. Similar results have been reported in studies conducted in Brazil [30] and the United States [31], which examined associations between demographic variables and diet quality as measured by dietary indices.

Participation in a food assistance program was associated with improved diet quality among women, highlighting its potential to reduce dietary inequalities. Food assistance programs improve diets by prioritizing healthy food access over mere caloric sufficiency. The Workers’ Food Program (WFP) exemplifies this mechanism, since by providing meals at the workplace planned by nutritionists to meet qualitative and quantitative nutritional parameters, the program ensures a balanced intake of nutrients, demonstrating how structured food policies directly improve the quality of workers’ diets. These programs aim to provide balanced meals in line with dietary guidelines or offer meal vouchers for direct food purchases, often coupled with educational initiatives to promote healthier eating practices. This finding supports the role of public policies, such as WFP, in fostering healthier eating habits among vulnerable populations, where women may be more likely to adopt healthy dietary practices when given access to supportive resources and nutrition information.

Anthropometric measures, such as BMI and waist circumference, showed no significant associations with diet quality, but a negative association with overweight among men was statistically significant. This finding aligns with some previous studies, particularly longitudinal studies that have demonstrated that diet quality influences weight gain and metabolic health over time [3]. The absence of associations with the anthropometric indices in this study may be due by the relatively homogeneous nature of the sample, the potential influence of unmeasured confounding variables, or sheer lack of statistical power.

The analysis of food choice dimensions highlights the importance of psychological and behavioral determinants in shaping dietary patterns. The results showed that prioritizing natural content was associated with higher diet quality in both sexes, while weight control emerged as a significant factor only among women. Previous research reports that weight control motivation is more pronounced among women, suggesting a stronger inclination towards healthy eating behaviors and, consequently, better diet quality [32]. For example, a large study conducted in China found that individuals engaging in weight control behaviors were more likely to be female and had higher diet quality scores [33]. Women often prioritize the relationship between diet and health, take primary responsibility for food preparation, and tend to consume more fruits, vegetables, and fiber while limiting fat intake [34–36]. These patterns may stem from greater health awareness, more developed nutritional knowledge, and a clearer understanding of what constitutes a healthy diet [37].

The analysis of the association of food choice motivations to specific dimensions of the DQI-I helps in the understanding of how different motivations influence different aspects of dietary intake. For instance, the “Health”, “Natural content”, “Weight control”, and “Familiarity” dimensions were significantly associated with both better “Variety” and “Adequacy” scores, suggesting that individuals consciously trying to eat healthily or manage their weight are actively incorporating a wider range of food groups. The “Natural content” motivation was significantly and positively associated with the “Moderation” component, a finding that suggests that a preference for natural, minimally processed foods can lead to a diet lower in harmful constituents. Conversely, the absence of any significant associations with the “General Balance” component reinforces the idea that achieving the correct macronutrient proportions is a more complex task, likely influenced by overall dietary patterns rather than a single conscious motivation. Understanding that the natural content of food influences dietary choices can guide the proposal and improvement of public policies to promote unprocessed foods, for example, through economic subsidies, supply regulation, and rigorous monitoring of marketing claims. Furthermore, restructuring local food environments by restricting fast-food sales near schools and encouraging the sale of fresh produce, coupled with clear labeling initiatives, can comprehensively support healthier food choices.

The strongest associations emerged from the analysis of the degree of food processing. Studies using the NOVA classification system have shown that ultra-processed foods are typically high in added sugars, unhealthy fats, and sodium, while being low in essential nutrients. Higher consumption of unprocessed foods and lower intake of ultra-processed foods were consistently associated with better diet quality, reinforcing the harmful impact of ultra-processed foods on dietary health [22]. The adverse effects of processed and ultra-processed foods are well documented [38–41].

The analysis of the relation of ultra-processed food consumption to DQI-I components presented in this paper, elucidates the specific mechanisms through which ultra-processed foods degrade dietary quality. The data reveal that UPF intake is not merely a marker of poor nutrition but actively compromises nearly every dimension of a healthy diet. The most pronounced and consistent negative associations were observed in the ‘Variety’ and ‘Moderation’ components. The strong inverse relationship with ‘Variety’ suggests that diets high in ultra-processed foods displace a diverse range of whole foods, leading to a monotonous intake pattern that is unlikely to meet the body’s needs for a broad spectrum of phytonutrients and essential compounds. Concurrently, the significant negative association with ‘Moderation’ directly reflects the well-documented nutritional profile of ultra-processed foods, which are typically engineered to be high in added sugars, unhealthy fats, and sodium, which are precisely the components the DQI-I seeks to limit.

The results of this study can be interpreted through the lens of the socio-ecological model, which posits that dietary behaviors are influenced by multiple levels of interaction, including individual, interpersonal, community, and societal factors [42]. At the individual level, factors such as age, sex, and food choice motivations (such as natural content and weight control) shape dietary patterns. Interpersonal factors, including marital status and family structure, may also influence diet, although these were not significant in the current study. Community-level factors, such as participation in food assistance programs, emerged as important determinants, highlighting the role of public policies in shaping dietary habits. At the societal level, broader economic conditions and food environment factors, such as income and food processing trends, also influence diet quality.

The study identified key modifiable factors influencing diet quality. Sex-specific trends, such as the influence of age and food assistance among females, suggest that tailored public health interventions may be necessary. Notably, the strong association between ultra-processed food consumption and poorer diet quality highlights the need for public health strategies aimed at reducing the intake of these products. Policies such as front-of-pack labelling, taxes on sugar-sweetened beverages, and nutrition education programs could serve as effective interventions [21]. Furthermore, addressing socioeconomic and gender-specific factors may enhance the effectiveness of nutrition-related policies and programs. The observed positive impact of food assistance initiatives, particularly the Workers’ Food Program (WFP), on diet quality suggests that expanding such efforts could help reduce dietary inequalities.

This study has several strengths, including the use of a representative sample of manufacturing workers, validated dietary assessment methods (such as the 24-hour recall and DQI-I), and a comprehensive analysis of socio-demographic, behavioral, and food processing-related factors. The inclusion of both men and women enabled sex-specific analyses, which revealed meaningful differences in the determinants of dietary patterns.

However, the study also has limitations. Its cross-sectional design does not allow for causal inferences, and reliance on self-reported dietary data may introduce recall bias. Although the 24-hour recall method is widely used, it may not fully reflect habitual intake. In addition, the focus on a specific occupational group may limit the generalizability of the findings to broader populations.

Future research should investigate the long-term health outcomes associated with diet quality in similar populations and assess the effectiveness of targeted interventions to promote healthier dietary patterns.

## Conclusions

This study provides valuable insights into the determinants of diet quality in a low-income Brazilian population of manufacturing workers. The findings highlight the importance of socio-demographic characteristics, food choice motivations, and levels of food processing in shaping dietary patterns. The study contributes to the global literature on diet quality by presenting evidence from an LMIC context, where the double burden of malnutrition, comprising both undernutrition and obesity, is becoming increasingly prevalent. Addressing these challenges requires a multi-sectoral approach that takes into account the complex interactions between socio-economic, behavioral, and environmental factors that influence dietary habits.

## Supporting information

S1 File Study datafile. (XLSX)
